# Hybrid Management of Stenosed Bicuspid Aortic Valve with Associated Coarctation of Aorta in an Adult: A Case Report

**DOI:** 10.1186/1749-8090-10-S1-A35

**Published:** 2015-12-16

**Authors:** Bipin Chandra, Surendra K Agarwal, Shantanu Pande, Gauranga Majumdar, Navneet K Srivastav, Aditya Kapur

**Affiliations:** 1 Department of Cardio Vascular and Thoracic Surgery, Sanjay Gandhi Post Graduate Institute of Medical Sciences, Lucknow, Uttar Pradesh, 226014, India; 2 Department of Cardiology, Sanjay Gandhi Post Graduate Institute of Medical Sciences, Lucknow, Uttar Pradesh, 226014, India

## Background/Introduction

Isolated coarctation of aorta is usually diagnosed and treated surgically in most of patients during childhood, but optimal management strategies for adult patients with coarctation of aorta associated with stenosed bicuspid aortic valve are controversial.

## Aims/Objectives

We aim to treat such rare patients successfully by less invasive two staged hybrid management.

## Method

We report a case of 18 years old female with history of dyspnoea on exertion since 4 years, with complaints of headache and bilateral lower limb claudication, clinical findings revealed no characteristic clinical feature of Turner syndrome, on examination femoral pulses were delayed and diminished bilaterally, laboratory examination were unremarkable with negative karyotyping for Turner syndrome, chest x-ray showed enlarged cardiac silhouette with rib notching, echocardiography revealed severely stenosed bicuspid aortic valve with left ventricular hypertrophy, descending aortogram showed post ductal coarctation and collaterals, pressure analysis revealed gradient of 70 mmHg across coarctation. She underwent successful hybrid approach with percutaneous catheter balloon coarctoplasty (PCBC) with stenting in the first stage, followed by surgery for aortic valve replacement in the second stage.

## Results

Post operatively she was asymptomatic and had uneventful recovery after each stage of management, post-operative echocardiography findings were within acceptable limits, and at 6 months follow up the mean echocardiographic aortic arch gradient was within acceptable limits.

## Discussion/Conclusion

Two staged hybrid approach of (PCBC) with stenting for coarctation of aorta and aortic valve replacement for coexistent stenosed bicuspid aortic valve is a safe, less invasive and can be effective alternative to complete surgical approach, if done meticulously.

## Consent

Written informed consent was obtained from the patient for publication of this abstract and any accompanying images. A copy of the written consent is available for review by the Editor of this journal.

